# Neutrophil extracellular traps trigger alveolar epithelial cell necroptosis through the cGAS-STING pathway during acute lung injury in mice

**DOI:** 10.7150/ijbs.99456

**Published:** 2024-09-03

**Authors:** Han-Xi Sha, Yu-Biao Liu, Yan-Ling Qiu, Wen-Jing Zhong, Nan-Shi-Yu Yang, Chen-Yu Zhang, Jia-Xi Duan, Jian-Bing Xiong, Cha-Xiang Guan, Yong Zhou

**Affiliations:** 1 Department of Physiology, School of Basic Medical Science, Central South University, Changsha, Hunan 410078, China.; 2 Key Laboratory of General University of Hunan Province, Basic and Clinic Research in Major Respiratory Disease, Changsha, Hunan 410078, China.; 3 National Experimental Teaching Demonstration Center for Medical Function, Changsha, Hunan 410013, China.

**Keywords:** Acute lung injury, Neutrophil extracellular traps, Alveolar epithelial cells, Necroptosis, cGAS-STING, Endocytosis

## Abstract

Extensive loss of alveolar epithelial cells (AECs) undergoing necroptosis is a crucial mechanism of acute lung injury (ALI), but its triggering mechanism needs to be thoroughly investigated. Neutrophil extracellular traps (NETs) play a significant role in ALI. However, the effect of NETs on AECs' death has not been clarified. Our study found that intratracheal instillation of NETs disrupted lung tissue structure, suggesting that NETs could induce ALI in mice. Moreover, we observed that NETs could trigger necroptosis of AECs *in vivo* and *in vitro*. The phosphorylation levels of RIPK3 and MLKL were increased in MLE12 cells after NETs treatment (*P* < 0.05). Mechanistically, NETs taken up by AECs through endocytosis activated the cGAS-STING pathway and triggered AECs necroptosis. The expression of cGAS, STING, TBK1 and IRF3 were increased in MLE12 cells treated with NETs (*P* < 0.05). Furthermore, the cGAS inhibitor RU.521 inhibited NETs-triggered AECs necroptosis and alleviated the pulmonary damage induced by NETs in mice. In conclusion, our study demonstrates that NETs taken up by AECs *via* endocytosis can activate the cGAS-STING pathway and trigger AECs necroptosis to promote ALI in mice. Our findings indicate that targeting the NETs/cGAS-STING/necroptosis pathway in AECs is an effective strategy for treating ALI.

## Introduction

Acute lung injury (ALI) and its severe form, acute respiratory distress syndrome (ARDS), are non-cardiogenic forms of diffuse lung injury and progressive respiratory failure caused by multiple factors both within and outside the lungs 1, with clinical manifestations of bilateral lung infiltration, refractory hypoxemia, and non-cardiogenic pulmonary edema 2. At present, the pathogenesis of ALI/ARDS is not fully understood and lacks specific treatment. The mortality of hospitalized ARDS patients can be as high as 35%-45% 3. Therefore, it is of great theoretical and clinical significance to investigate the pathogenesis of ALI/ARDS.

Alveolar epithelial cells (AECs) are essential parenchymal cells that maintain the structural and functional integrity of the lungs 4. AECs are easily damaged due to long-term exposure to the environment containing pathogenic factors 5, 6. Extensive loss of AECs is one of the central pathogenic mechanisms of ALI 7. Various irritants, such as viruses and bacteria, can cause damage to AECs, resulting in the loss of AECs and disruption of the lung epithelial barrier 8, 9. Damaged AECs release damage-associated molecular molecules (DAMPs) to activate alveolar macrophages, amplify inflammatory responses, and participate in the development of ALI/ARDS 10. Infection with the 2019 novel coronavirus (2019-nCoV) damages the structure and function of AECs, ultimately leading to massive loss of AECs and exacerbating ALI 11. Therefore, it is vital to target the mechanism of AECs injury during ALI/ARDS and find effective intervention strategies.

AECs are lost in a variety of ways, including apoptosis, ferroptosis, necroptosis, and so on 12. Necroptosis, a programmed cell death triggered by disturbance of a cell's homeostasis, is dependent on the activation of receptor-interacting protein kinase 3 (RIPK3) and the mixed lineage kinase domain-like protein (MLKL) 13. Necroptosis is an inflammatory mode of death that triggers an inflammatory cascade amplification response 14. Our and other's studies have shown that necroptosis is the primary mode of death of AECs in lipopolysaccharide (LPS)-induced ALI mice 15-17. However, the mechanism that triggers necroptosis in AECs during ALI is not fully understood.

Neutrophil overactivation is one of the key pathological features of ALI/ARDS 18. Neutrophil extracellular traps (NETs) are extracellular network structures released during NETosis, a suicidal inflammatory mode of neutrophil death 19. NETs have DNA skeleton and are embedded with a series of proteins with bactericidal and permeability-increasing effects such as citrullinated histone 3 (CitH3), myeloperoxidase (MPO), and neutrophil elastase (NE) 20, 21. Studies have shown that NETs can be harmful factors in damaging lung parenchymal cells and lung immune cells, triggering inflammatory cascade reactions, and exacerbating lung injury. NETs induce pulmonary microvascular endothelial cell death, causing alveolar and capillary damage and promoting the development of ALI/ARDS 22, 23. NETs promote LPS-induced pyroptosis of alveolar macrophages by activating the macrophage absent in melanoma 2 (AIM2) inflammasome 24. However, the role of NETs in necroptosis of AECs during ALI and its molecular mechanism has not been reported.

NETs are DNA-based skeleton network structures released into the extracellular space by neutrophils 19. It has been reported that NETs can be phagocytosed by macrophages and other myeloid cells, thereby activating the cGAS-STING pathway and inducing the production of IFN-γ 25. However, it is unclear whether AECs can take up NETs by endocytosis. The cGAS-STING signaling pathway is an innate immune pathway discovered in 2013, activation of which triggers an autoinflammatory response 26. Cyclic GMP-AMP synthase (cGAS) catalyzes GTP and ATP synthesis of cyclic GMP-AMP (cGAMP) after recognizing double-stranded DNA (dsDNA) in the cytoplasm 27. cGAMP binds and activates the stimulator of interferon genes (STING), which promotes the translocation of STING to the Golgi apparatus. Subsequently, STING can activate downstream tank-binding kinase 1 (TBK1) and phosphorylated interferon regulatory factor 3 (IRF3). Cytoplasmic IRF3 dimer enters the nucleus after phosphorylation, leading to the production of downstream interferon (IFN) and tumor necrosis factor (TNF), which are involved in a variety of pathological processes 28, 29.

In this study, we found that NETs induced necroptosis of AECs and promoted ALI in mice. Mechanistically, NETs could be taken up by AECs through endocytosis, which activated the cGAS-STING pathway, induced necroptosis of AECs, and promoted ALI in mice. Our study suggests that targeting the cGAS-STING pathway in NETs-mediated necroptosis of AECs may provide new ideas for the prevention and treatment of ALI/ARDS.

## Materials and Methods

### Animal experiments

The 8-week-old male C57BL/6J mice were provided by Hunan SJA Laboratory Animal Co., Ltd (Hunan, China) and housed in the pathogen-free facility of Central South University. All animal protocols were approved by the Ethics Committee of the Basic Medical School of Central South University (2023-KT026).

### Animal treatments

To obtain the dosage of NETs for intratracheal injection in mice, we first gave mice intratracheal injection of LPS (5 mg/kg, from *E. coli O111:B4*, Sigma-Aldrich, MO, USA) to construct a classical ALI model. Mice were killed 12 h after injection of LPS, and serum and bronchoalveolar lavage (BALF) were collected. An ELISA kit was used to measure the amount of MPO-DNA in serum and BALF of mice to obtain the amount of intratracheally injected with NETs in mice.

C57BL/6J mice were randomly divided into Control, NETs, and NETs+RU.521 groups (RU.521: a specific inhibitor of cGAS). Mice were anesthetized with pentobarbital sodium. Control mice were injected with saline. Mice in the NETs group received intratracheal injections of extrinsic NETs. To assess the role of the cGAS-STING pathway in NETs-induced necroptosis of mouse AECs, mice in the NETs+RU.521 group were injected with RU.521 (5 mg/kg, RU.521 dissolved in saline solution, MedChemExpress, USA) intraperitoneally 2 h before the intratracheal injection of NETs. Exogenous NETs were extracted from mouse bone marrow neutrophils. All mice were sacrificed 12 h after injection of exogenous NETs. Lung tissue, serum, and BALF were collected and stored for subsequent experiments.

### Neutrophil stimulation and isolation of NETs

Neutrophils were isolated from whole blood using Percoll (Cytiva, USA) with density gradient centrifugation. Mouse bone marrow neutrophils were isolated using Ly6G (mouse neutrophil-specific surface marker) coated beads (Miltenyi Biotec, USA). Neutrophils were cultured using RPMI 1640 (Gibco, USA) containing 10% heat-inactivated bovine calf serum (BCS, Gibco, USA) at 37°C and then seeded at a density of 1×10^7^ cells per mL in 6-well plates. After one hour of placement, neutrophils were treated with 15 ng/mL of phorbol 12-myristate 13-acetate (PMA, Sigma-Aldrich) for 4 h at 37°C and 5% CO_2_. Gently suck out and discard the cell culture medium so that the NETs and neutrophil layers stick to the bottom. Cells were washed twice with ice-cold PBS to extract all adhesions from the bottom of each petri dish. NETs were collected in 15-mL tubes by scraping and centrifuged at 450 g for 10 min at 4 ℃. The supernatant was divided into 1.5 mL microcentrifuge tubes and centrifuged at 18,000 g for 15 min at 4 ℃. The supernatant was discarded, and the remaining product was re-suspended with ice-cold PBS. The DNA concentration of supernatants containing NETs was measured using a NanoDrop 2000 Spectrophotometer (Thermo Fisher Scientific). Use immediately or store frozen at -80°C.

### H&E staining and inflammatory injury score

Twelve hours after the NETs injection, the lungs of the mice were fixed with a 4% neutral buffered formaldehyde solution. Lung slices were stained with H&E (Solarbio, China, Beijing) 30. Images were captured using Pannoramic Scan (3Dhistech, Hungary, Budapest). Histopathological analysis of paraffin-embedded lung tissue was performed on lung sections stained with H&E using standard procedures. A numerical inflammatory score was used to semiquantitatively evaluate the degree of morphological alterations (neutrophils in the alveolar space, bleeding, hyaline membranes, pertinacious debris filling the airspaces, and septal thickening) 30, 31. The mean score was considered the inflammation score (0-4) and was determined by three independent pathologists 32. The inflammatory injury score was performed in a double-blind fashion.

### Cell culture and treatment

Mouse alveolar epithelial cell lines (MLE12) were obtained from ATCC (USA, CRL-2110) and cultured in Dulbecco's modified Eagle's medium/F12 (Gibco, USA) supplemented with 2% BCS at 37 ℃ in an air incubator of 95% air and 5% CO_2_. Cells were planted into the 12-well plate (1×10^5^ cells/well) and grown for 24 h until the cell density reached 60%. Cells were stimulated with 500 ng/mL NETs for 12 h. To evaluate the role of dsDNA in NETs-induced cGAS-STING pathway activation and necroptosis of MLE12 cells, we treated the cells with dsDNA degrading agent (DNase I, 10 µg/mL Roche, Switzerland) 30 min before NETs stimulation. To assess the role of the cGAS-STING pathway in NETs-induced necroptosis of MLE12 cells, we treated the cells with the cGAS inhibitor (RU.521, 10 µM, MedChemExpress, USA) or the STING inhibitor (H151, 5 µM, MedChemExpress, USA) 1 h before NETs stimulation. To elucidate the role of the endocytosis pathway in mediating NETs-induced necroptosis of MLE12 cells, we treated the cells with an inhibitor of endocytosis (Dynasore, 80 µM, MedChemExpress) for 30 min before the stimulation of NETs.

### Cell counting kit 8 assay

The survival rate of MLE12 cells was determined using the Cell Counting Kit 8 (CCK-8, Glpbio, USA). MLE12 cells were inoculated into a 96-well plate (2.5×10^3^ cells/well). Cells were cultured in a CO_2_ incubator at 37 ℃ for 6 h. We treated the cells with different factors and continued to culture them for 12 h. Then, 10 µL of CCK8 solution was added to each well and incubated for 1 to 4 h. Absorbance at 450 nm was measured using an enzyme-labeled spectrophotometer (MIULAB, China).

### LDH release assay

LDH released from MLE12 cells after treatment was measured using the LDH Cytotoxicity Assay Kit (Nanjing JianCheng Bioengineering Institute, China). Cells were plated in the 24-well plate (5×10^4^ cells/well) and grown for 24 h until the cell density reached 60%. The cell culture medium supernatant was collected, and the experiment was performed according to the kit instructions. Absorbance at 450 nm was measured using an enzyme-labeled spectrophotometer (MIULAB, China).

### Western blot

RIPA lysate (containing a protease inhibitor, Solarbio, China) was added to the lung tissue or cells. The samples were completely lysed at 4 ℃ for 30 min and centrifuged at 12,000 g for 10 min. The supernatant was absorbed, and the total protein concentration was determined using a bicinchoninic acid assay (BCA, Ding Guo Prosperous, China). After denaturation at 95 °C for 10 min, 30 μg protein was loaded on SDS-PAGE gels for electrophoresis. Proteins were transferred to PVDF membranes and blocked with 5% skim milk or bovine serum albumin (BSA, BioFroxx, China) for 1 h. The primary antibody was incubated overnight at 4 ℃. The next day, the secondary antibody was incubated with TBST wash film at room temperature for 1 h. After rewashing the PVDF membrane with TBST, the ECL luminescence solution was added for imaging. Western blotting was performed as previously described 33. Statistical analysis was performed using Image Lab software. The antibodies used in this study are listed in Table 1.

### Real-time PCR

Total RNA was extracted from cells using the RNAiso Plus kit (Takara, Kusatsu, Japan). The purity and concentration of the extracted RNA were determined using the NanoDrop 2000. RNA was reverse transcribed into cDNA using the PrimeScript™ RT reagent Kit (Takara, Kusatsu, Japan). Real-time PCR was performed in a CFX96 Touch™ instrument. The relative expression of genes was calculated using the 2^-ΔΔCt^ method, as previously described. The primers used in this study were synthesized by Tsingke Biotechnology (Beijing, China). The primer sequences used in the study are shown in Table 2.

### Immunofluorescence

Lung tissue sections were hydrated to facilitate antigen retrieval. The sections were placed in a solution of 0.01 M sodium citrate buffer (pH 6.0), which was heated in an autoclave and boiled for 2 min. The solution was cooled naturally for 30 min. The sections were incubated with 3% H_2_O_2_ for 15 min to inactivate endogenous peroxidase. Next, 5% BSA was occluded for 30 min. Lung tissue sections were incubated with anti-SP-C (1:2000) for 1 h at 37℃. Then, lung tissue sections were incubated with Polymer-HRP anti-mouse/rabbit universal secondary antibody (AiFang Biological, Hunan, China) for 30 min at room temperature. Next, lung tissue sections were reacted directly by dropwise addition of ready-to-use TYR-520 fluorescent dye (green light) from the Three-color Fluorescence kit (AiFang Biological, Hunan, China) based on the tyramide signal amplification (TSA) technology for 10 min at room temperature. After that, lung tissue sections were re-subjected to the following procedures, including antigen retrieval, inactivation of endogenous peroxidase, and blocking. Lung tissue sections were incubated with primary antibodies, including anti-cGAS (1:250) and anti-MLKL (1:200) at 4°C overnight. The next day, after washing with PBS buffer, lung tissue sections were incubated with Polymer-HRP anti-mouse/rabbit universal secondary antibody for 30 min at room temperature. Next, lung tissue sections were reacted directly by dropwise addition of ready-to-use TYR-570 fluorescent dye (red light) from the Three-color Fluorescence kit for 10 min at room temperature. Nuclei were stained with DAPI (Invitrogen, USA) for 10 min. Sections were blocked with an anti-fluorescence quencher (Solarbio, China). The antibodies used in the study are shown in Table 1.

The cells were washed with PBS and fixed with 4% paraformaldehyde (Biossci, China) for 15 min. The cells were next permeabilized with 0.1% Triton X-100 (Beyotime, China) for 8 min and blocked with 1% BSA for 30 min at room temperature. Primary antibodies were incubated at 4°C overnight. Primary antibodies included anti-MLKL (1:500, Proteintech), anti-RIPK3 (1:200), anti-STING (1:200), anti-cGAS (1:250), anti-IRF3 (1:50), anti-NF-κB (1:200) and anti-MPO (1:200). The next day, fluorescent secondary antibodies were incubated for 1 h at room temperature, protected from light. Phalloidin was incubated for 30 min at room temperature and protected from light. After washing with PBS, cells were stained with DAPI (Solarbio, China) for 10 s. Images were acquired with a Laser Scanning Confocal Microscope (Leica SP8, Germany). The antibodies used in the study are shown in Table 1.

### Cytokine detection

TNF-α contents in the cell culture supernatant were measured using appropriate enzyme-linked immunosorbent assay (ELISA) kits according to the manufacturer's protocols (Invitrogen, Thermo Fisher Scientific, USA).

### RNA-seq analysis

Total RNA was extracted using an RNAiso Plus kit (Takara, Kusatsu, Japan). Following cluster generation, library preparations were sequenced on the Illumina platform by Majorbor (Shanghai, China), resulting in the generation of raw reads. DESeq2 was employed for gene expression analysis of the different groups. Genes with *P* < 0.05 were identified as differentially expressed genes (DEGs). The data were analyzed through the free online platform provided by Majorbio Cloud Platform (www.majorbio.com). The RNA-seq data of AECs (MLE12) utilized in this study are available from the corresponding author upon reasonable request.

### Statistical analysis

All values are expressed as the mean ± standard deviation of at least three independent experiments. Depending on the experimental design, statistical significance was determined using the unpaired *t*-test or ANOVA. All statistical tests were analyzed using GraphPad Prism 9.0 (San Diego, CA, USA). *P* < 0.05 indicated a significant difference.

## Results

### NETs induce necroptosis of AECs and promote ALI in mice

**Figure 1:**
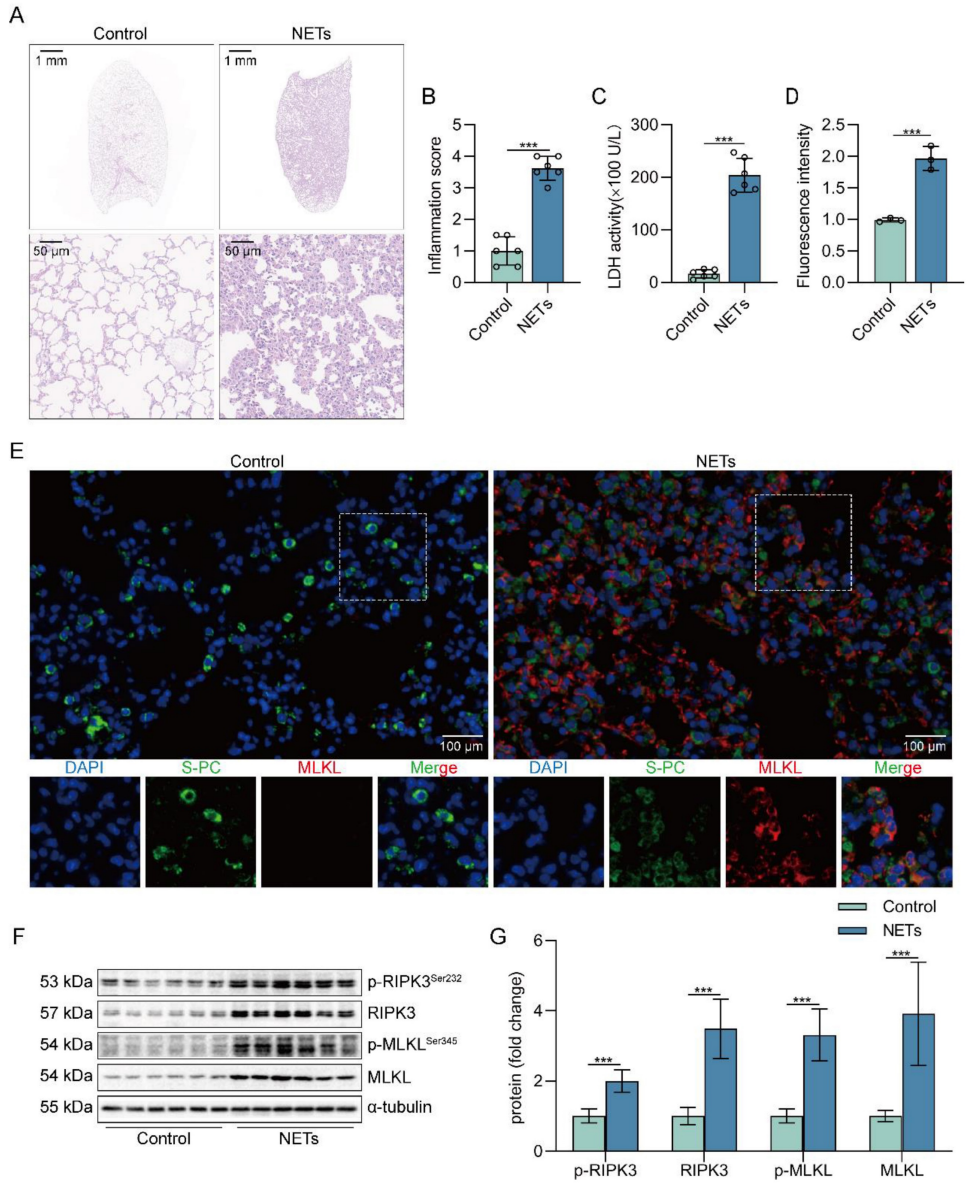
** NETs induce necroptosis of AECs and promote acute lung injury in mice.** C57BL/6J mice were intraperitoneally injected with NETs (3 µg/pcs) for 12 h. **A** H&E staining of lung tissue structure of mice stimulated with NETs for 12 h (bar=50 µm). **B** The inflammatory injury score was performed in a double-blind fashion (*n*=6). **C** LDH levels in the serum of mice stimulated with NETs for 12 h (*n*=6). **D-E** Immunofluorescence staining and confocal microscopy were used to determine the localization of MLKL and SP-C (bar=100 µm). Green fluorescence represents alveolar epithelial cell marker protein SP-C, red fluorescence represents necroptosis key protein MLKL, and DAPI blue fluorescence represents the nucleus. **F-G** The result of the western blot showed elevated expression of the necroptosis-related proteins RIPK3 and MLKL and increased levels of phosphorylated MLKL and RIPK3 (*n*=6). Data are expressed as the mean ± SD. Comparisons between the two groups were made with an unpaired* t*-test. ***P < 0.001.

To determine whether NETs can induce lung tissue damage, mice were intratracheally injected with NETs. The dosage of NETs injected for intratracheal injection in mice was calculated to be 150 µg/kg by ELISA (Figure S1). Histological study showed that the lung tissue structure of mice stimulated with NETs for 12 h was significantly damaged, with alveolar fusion and collapse, thickening of alveolar septa, and accompanied by a large number of inflammatory cell infiltration (Figure 1A). Moreover, NETs injection significantly increased the inflammation score of lung tissue in mice (Figure 1B), suggesting that NETs could cause pathological damage to lung tissue in mice. LDH levels in serum were significantly increased in NETs-stimulated mice compared with normal mice (Figure 1C). In addition, the number of necroptosis AECs (SP-C^+^MLKL^+^) was significantly increased in the lung tissue of mice receiving NETs (Figure 1D-E), suggesting that AECs in the lungs of NETs-treated mice underwent necroptosis. Furthermore, NETs treatment significantly increased the levels of RIPK3, MLKL, and their phosphorylation in mouse lung tissue (Figure 1F-G). Taken together, these data demonstrate that NETs induce necroptosis in AECs in mice and promote ALI.

### NETs induce necroptosis of AECs *in vitro*

**Figure 2:**
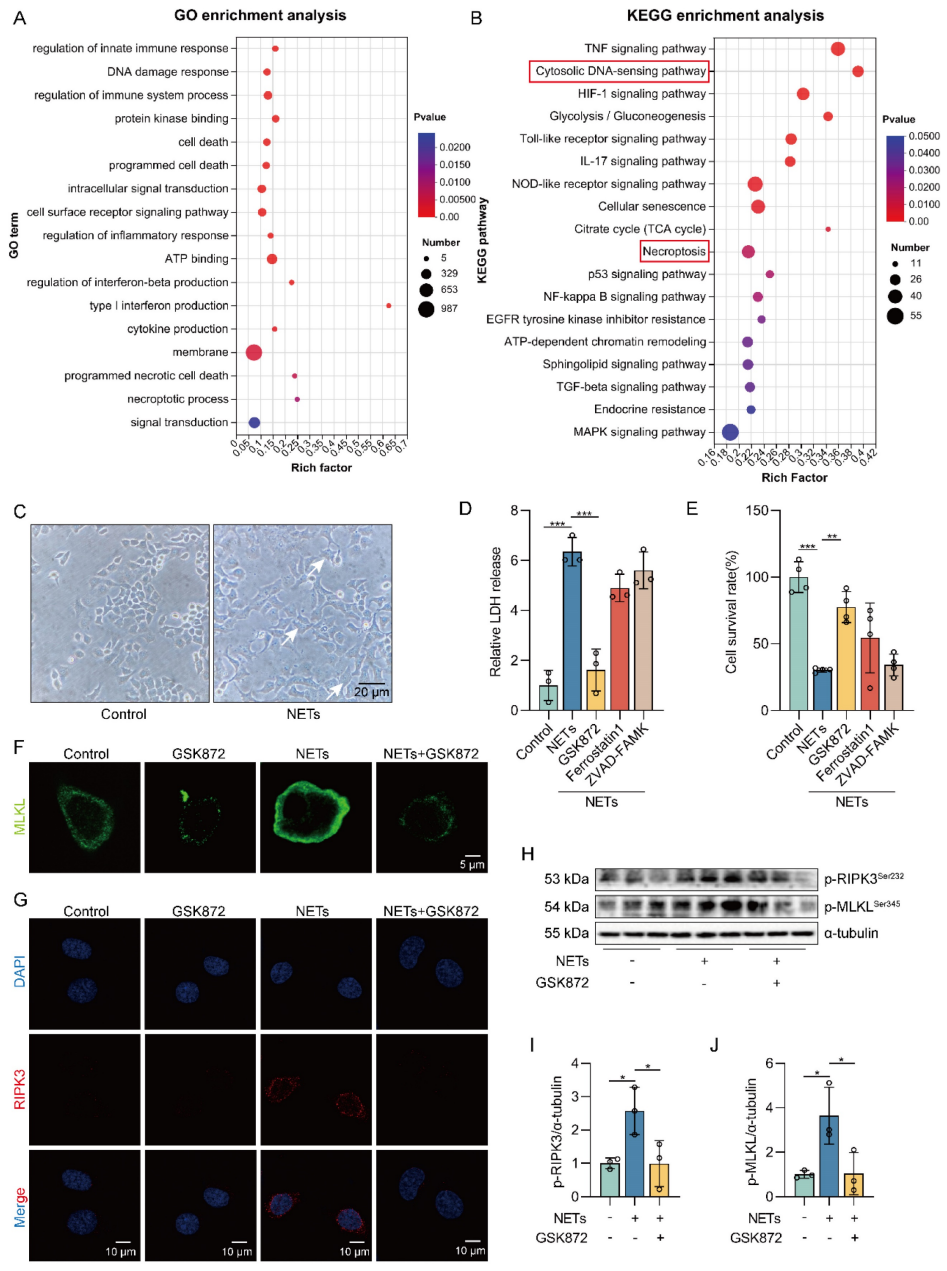
** NETs induce necroptosis of AECs *in vitro*.** MLE12 cells were treated with NETs (500 ng/mL) for 12 h after GSK872 (10 μM), Z-VAD-FMK (50 μM), or ferrostatin-1 (1 μM) intervention 1 h earlier. **A-B** GO and KEGG analyses. GO analyses demonstrate the changes in molecular function, cellular component, and biological process in NETs-treated MLE12 cells; KEGG analyses demonstrated activation of major pathways in NETs-treated MLE12 cells. **C** Representative images of control and NETs-treated MLE12 cells (bar=20 µm). **D** Evaluation of MLE12 cell mortality by LDH release assay (*n*=3). **E** MLE12 cell viability was evaluated by CCK-8 assay (*n*=4). **F** Immunofluorescence staining for MLKL (green) (bar=5 µm). **G** Immunofluorescence staining for RIPK3 (red) (bar=10 µm). **H-J** The phosphorylation levels of RIPK3 and MLKL in MLE12 cells (*n*=3). Data are expressed as the mean ± SD. Differences among multiple groups were performed using ANOVA. *P < 0.05, **P < 0.01, and ***P < 0.001.

We treated MLE12 cells with different concentrations of NETs (100, 250, 500, and 750 ng/mL) and found that the survival rate of MLE12 cells decreased as the NETs concentration increased (Figure S2A). So, we treated MLE12 cells with NETs (500 ng/mL) for 12 h for the following experiments. To assess whether NETs can induce necroptosis of AECs *in vitro*, we performed RNA-seq analysis on MLE12 cells treated with or without NETs. Volcano plot results showed significant differences in gene expression between the control group and the NETs-treated group (Figure S2B). Gene Ontology (GO) and KEGG pathway enrichment analyses showed that the necroptosis signaling pathway was activated in NETs-treated MLE12 cells (Figure 2A-B).

Then, we observed that the cells in the NETs-treated group showed significant changes in cell morphology, with cell swelling and increased intracellular contents (Figure 2C). We found an increase in LDH release as well as a decrease in cell viability in NETs-treated MLE12 cells, which could be significantly improved by using the necroptosis inhibitor (GSK872). In contrast, no significant increase in MLE12 cell survival was observed with the use of ferroptosis inhibitor (Ferrostatin1) and apoptosis inhibitor (ZVAD-FAMK) (Figure 2D-E). These results suggest that necroptosis plays an essential role in the injury of AECs induced by NETs. In addition, NETs induced translocation of the trimerized MLKL to the plasma membrane to form pores, causing MLE12 cells to swell (Figure 2F). Meanwhile, we observed that NETs increased the expression of RIPK3 (Figure 2G). Similarly, the phosphorylation levels of RIPK3 and MLKL were also increased in MLE12 cells after NETs treatment (Figure 2H-J). All of these data imply that NETs induce necroptosis of AECs *in vitro*.

### The DNA skeleton of NETs is the main factor that triggers the necroptosis of AECs

**Figure 3:**
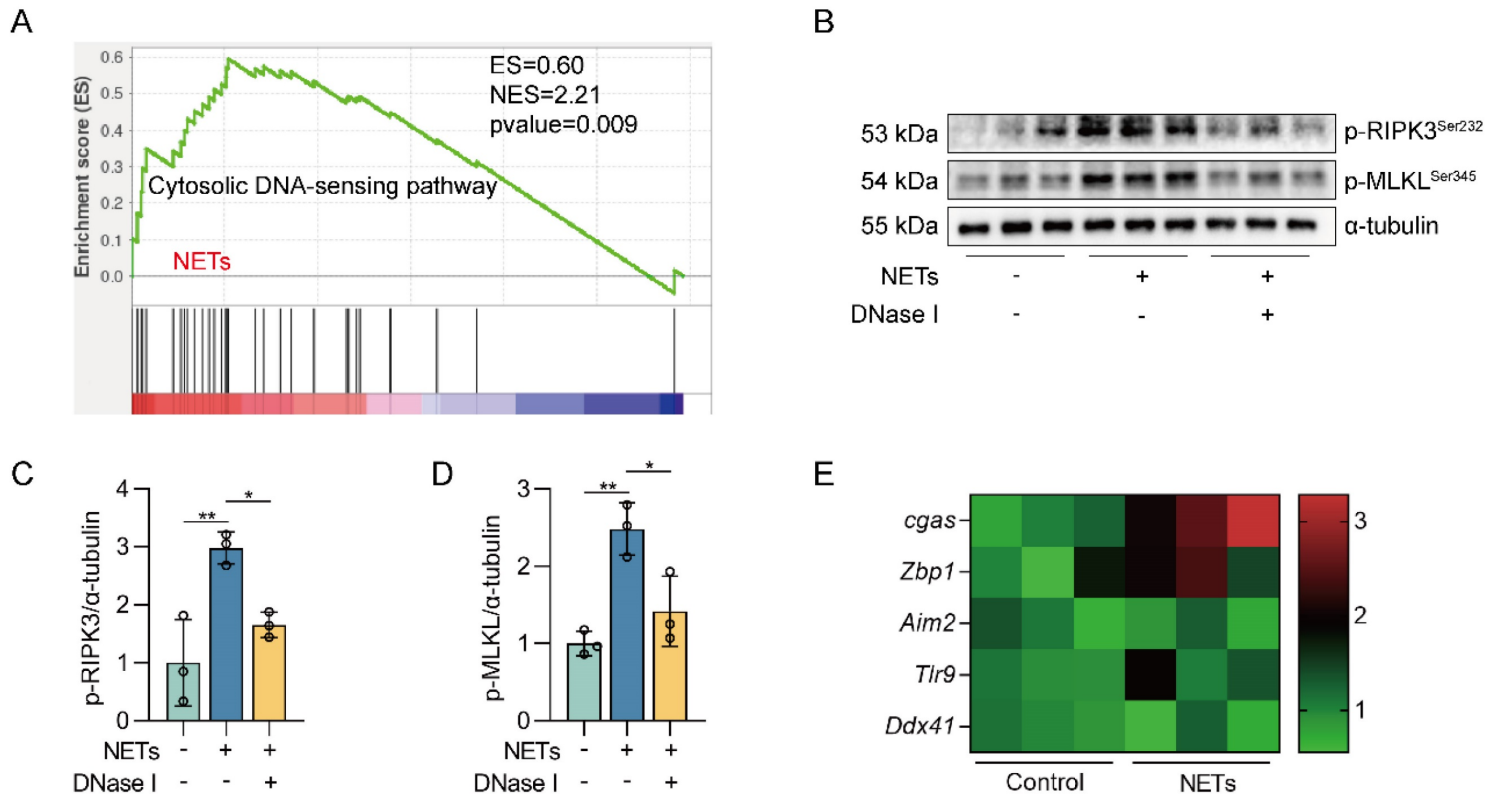
** The DNA skeleton of NETs is the main factor that triggers the necroptosis of AECs.** MLE12 cells were treated with NETs (500 ng/mL) for 12 h after DNase I (10 µg/mL) intervention 30 min earlier. **A** GSEA analysis of gene expression shows that the cytosolic DNA-sensing pathway was upregulated in NETs-treated MLE12 cells compared to control cells. **B-D** The phosphorylation levels of MLKL and RIPK3 in MLE12 cells were detected by western blot (*n*=3). **E** The mRNA expression levels of PRRs with identifiable DNA were determined by RT-PCR (*n*=3). Data are expressed as the mean ± SD. Differences among multiple groups were performed using ANOVA. *P < 0.05 and **P < 0.01.

We next investigated how NETs triggered necroptosis of AECs. GO and KEGG pathway enrichment analyses showed that the cytosolic DNA-sensing pathway was activated in NETs-treated MLE12 cells (Figure 2A-B). From GSEA analysis of transcriptome sequencing data, we found that the cytosolic DNA-sensing pathway was activated in NETs-treated MLE12 cells compared to control cells (Figure 3A). We then used DNase I pretreatment to degrade the DNA skeleton components in NETs. We found that DNase I significantly inhibited NETs-induced necroptosis in MLE12 cells (Figure 3B-D). These results suggest that the cytosolic DNA-sensing pathway plays a critical role in NETs-induced necroptosis and that dsDNA is an essential mediator of NETs-induced necroptosis in AECs.

DNA released into the extracellular space often functions as DAMPs in combination with corresponding pattern recognition receptors (PRRs) 34. The known DNA-based PRRs mainly consist of toll-like receptor 9 (TLR9), Z-DNA binding protein 1 (ZBP1), absent in melanoma 2 (AIM2), and cGAS 35. Compared with control cells, we observed the most significant increase in *cgas* gene expression in MLE12 cells treated with NETs (Figure 3E), suggesting that NETs released by neutrophils may function by binding to intracellular cGAS.

### NETs activate the cGAS-STING pathway of AECs *in vitro*

**Figure 4:**
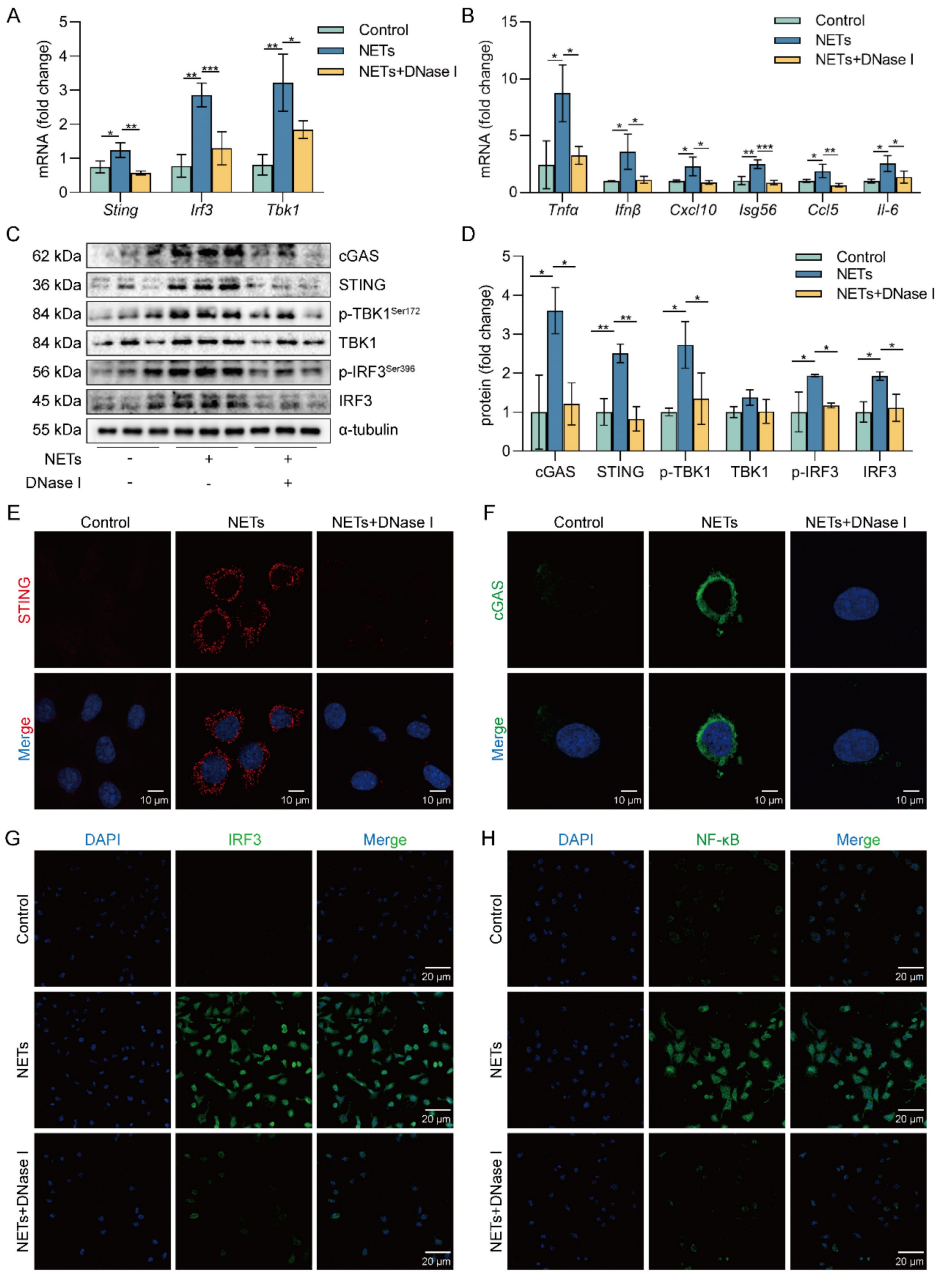
** NETs activate the cGAS-STING pathway *in vitro*.** MLE12 cells were treated with NETs (500 ng/mL) for 12 h after DNase I (10 µg/mL) intervention 30 min earlier. **A** The expression level of cGAS-STING pathway-related genes in MLE12 cells was detected by RT-PCR (*n*=3)*.* **B** The expression level of pro-inflammatory factors downstream of the cGAS-STING pathway in MLE12 cells was detected by RT-PCR (*n*=3). **C-D** The expression and phosphorylation levels of cGAS-STING pathway-related proteins in MLE12 cells were detected by western blot (*n*=3).** E** The expression of cGAS in MLE12 cells was detected by immunofluorescence (green) (bar=10 µm). **F** The perinuclear translocation of STING in MLE12 cells was detected by immunofluorescence (red) (bar=10 µm). **G** The nuclear translocation of IRF3 in MLE12 cells was detected by immunofluorescence (green) (bar=20 µm). **H** The nuclear translocation of NF-κB in MLE12 cells was detected by immunofluorescence (green) (bar=20 µm). Data are expressed as the mean ± SD. Differences among multiple groups were performed using ANOVA. *P < 0.05, **P < 0.01, and ***P < 0.001.

cGAS is one of the primary sensors of cytoplasmic DNA that activates STING, thereby initiating type I interferon and inflammatory responses 36, 37. Heatmap results showed that the expression of pro-inflammatory factor genes was upregulated in NETs-treated MLE12 cells (Figure S3A). We also observed increased expression of cGAS-STING pathway-related genes (*Sting*, *Tbk1*, and *Irf3*) as well as pro-inflammatory factor genes downstream of the cGAS-STING pathway in MLE12 cells treated with NETs compared with the control group, while this effect of NETs was inhibited by DNase I (Figure 4A-B). Besides, DNase I significantly reduced TNF-α secretion, which was promoted by NETs in MLE12 cells (Figure S3B). We also observed that the expression of cGAS-STING pathway-related proteins, including cGAS, STING, TBK1, IRF3, and the phosphorylation levels of TBK1 and IRF3, were increased in MLE12 cells treated with NETs, while the effect of NETs was inhibited by DNase I (Figure 4C-D). In MLE12 cells, NETs stimulation triggered the perinuclear translocation of STING, which further promoted the nuclear translocation of IRF3 and NF-κB, and the effect of NETs was inhibited by DNase I (Figure 4E-H). Collectively, the above data suggest that NETs can induce cGAS-STING pathway activation in AECs. Importantly, the dsDNA of NETs is an essential agent for NETs to trigger activation of the GAS-STING pathway of AECs.

### Inhibition of the cGAS-STING pathway suppresses the NETs-induced necroptosis of AECs *in vitro*

**Figure 5:**
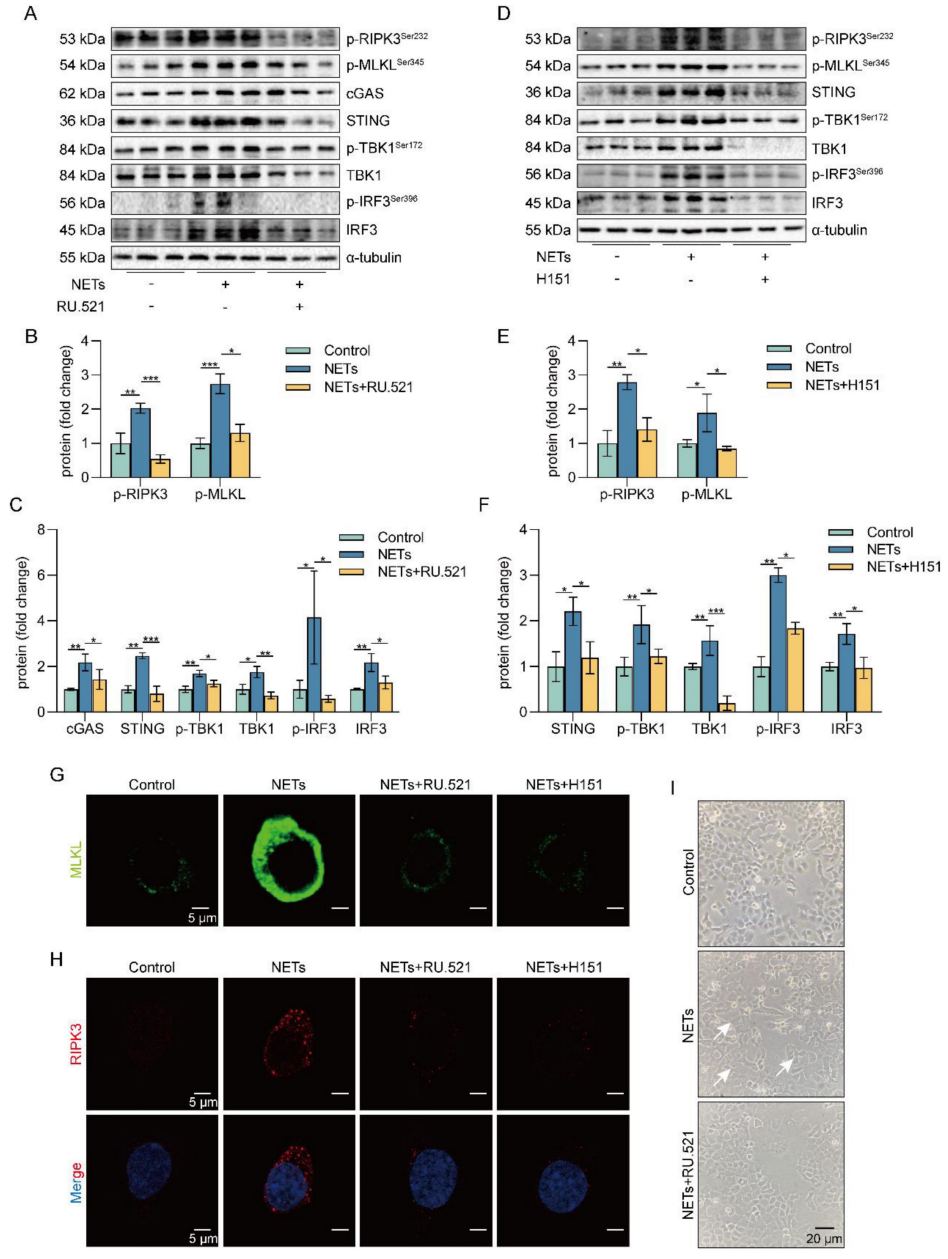
** Inhibition of the cGAS-STING pathway can suppress the AECs necroptosis induced by NETs *in vitro*.** MLE12 cells were treated with NETs (500 ng/mL) for 12 h after RU.521 (10 μM) or H151 (5 μM) intervention 1 h earlier.** A-F** The phosphorylation levels of necroptosis pathway-related proteins in MLE12 cells were detected by western blot (*n*=3). **G-H** Immunofluorescence staining for MLKL (green) and immunofluorescence staining for RIPK3 (red) (bar=5 µm) **I** Representative images of MLE12 cells from control, NETs, and NETs combined with RU.521 (bar=20 µm). Data are expressed as the mean ± SD. Differences among multiple groups were performed using ANOVA. *P < 0.05, **P < 0.01, and ***P < 0.001.

To investigate the role of the cGAS-STING pathway in the necroptosis of AECs induced by NETs, we used a cGAS inhibitor (RU.521) and a STING inhibitor (H151). We found that RU.521 alleviated the phosphorylation of necroptosis-related proteins, RIPK3 and MLKL, induced by NETs activation (Figure 5A-C). In addition, H151 also alleviated the phosphorylation levels of the necroptosis-related proteins, RIPK3 and MLKL, induced by NETs activation (Figure 5D-F). Besides, both RU.521 and H151 reduced NETs-induced trimerization of MLKL, which translocated to the plasma membrane to form pores in MLE12 cells (Figure 5G). Furthermore, the expression of RIPK3, a protein associated with NETs-induced necroptosis, was reduced by RU.521 or H151 (Figure 5H). RU.521 also significantly improved the morphological changes (cell swelling, increase in intracellular contents) induced by NETs (Figure 5I). Overall, inhibiting the activation of the cGAS-STING pathway can suppress the necroptosis of AECs induced by NETs *in vitro*.

### NETs are taken up by AECs through endocytosis and activate the cGAS-STING pathway to trigger the necroptosis of AECs *in vitro*

**Figure 6:**
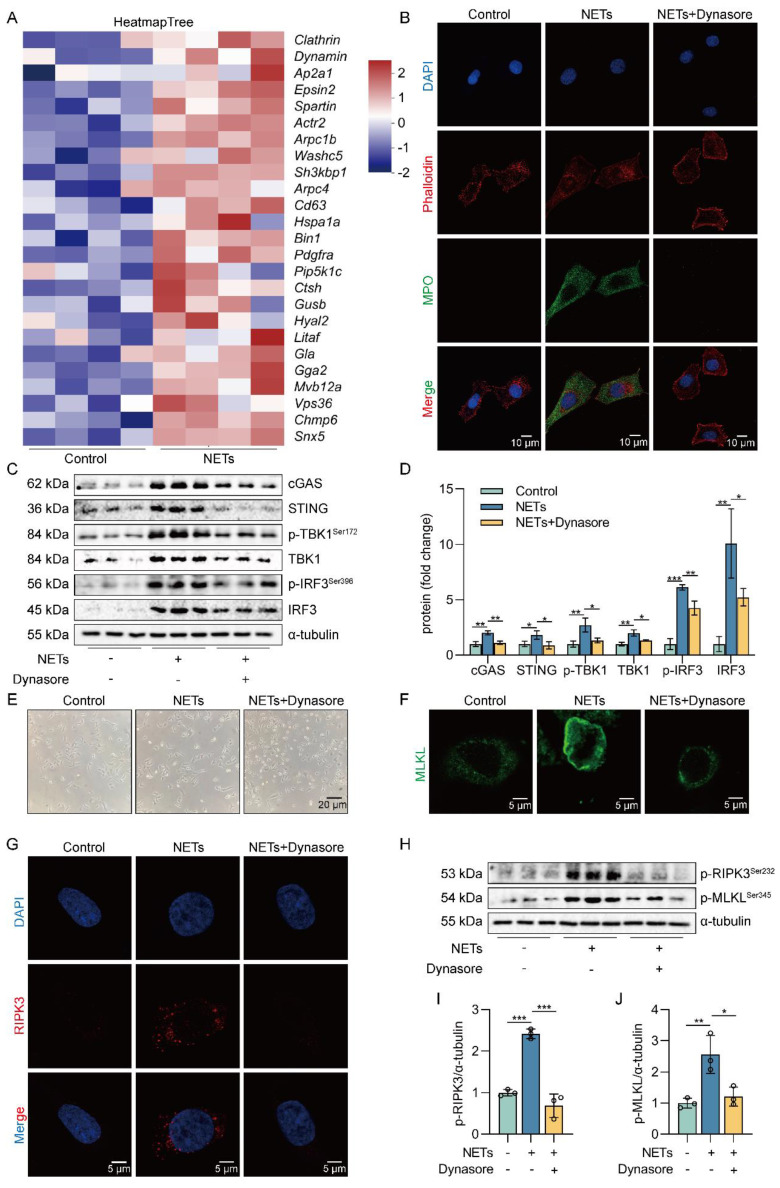
** NETs are taken up by AECs through endocytosis and activate the cGAS-STING pathway to trigger the necroptosis of AECs *in vitro*.** MLE12 cells were treated with NETs (500 ng/mL) for 12 h after Dynasore (80 μM) intervention 1 h earlier. **A** Heatmap analysis of RNA sequencing for clathrin and dynamin protein-related gene expression and lysosomal pathway-related gene expression in MLE12 cells as indicated*.* **B** The relative location of MPO in MLE12 cells was detected by immunofluorescence (bar=10 µm). MPO stands for NETs (green), and Phalloidin stands for the MLE12 cytoskeleton (red). **C-D** The expression and phosphorylation levels of cGAS-STING pathway-related proteins in MLE12 cells were detected by western blot (*n*=3). **E** Representative images of MLE12 cells from control, NETs, and NETs combined with Dynasore (bar=20 µm). **F** Immunofluorescence staining for MLKL (green) (bar=5 µm). **G** Immunofluorescence staining for RIPK3 (red) (bar=5 µm) **H-J** The phosphorylation levels of necroptosis pathway-related proteins in MLE12 cells were detected by western blot (*n*=3). Data are expressed as the mean ± SD. Differences among multiple groups were performed using ANOVA. *P < 0.05, **P < 0.01, and ***P < 0.001.

NETs are network structures released to the outside of the cell during neutrophil NETosis, while cGAS is a DNA recognition receptor present inside the cell 38, 39. Next, we investigated how extracellular NETs activate the intracellular recognition receptor cGAS. Heatmap results showed that the expression of clathrin and dynamin protein-related genes and lysosomal pathway-related genes were upregulated in NETs-treated MLE12 cells (Figure 6A). The results suggest that NETs may be swallowed by AECs through endocytosis. To further determine whether NETs were taken up by AECs through endocytosis, we used Dynasore, an endocytosis inhibitor. First, we found that NETs were located in the cytoplasm of MLE12 cells and were unable to enter MLE12 cells after using the endocytosis inhibitor Dynasore (Figure 6B). Then, we observed that compared with the NETs group, the expression of cGAS-STING pathway-related proteins, including cGAS, STING, TBK1, IRF3, and the phosphorylation levels of TBK1 and IRF3, were significantly reduced in MLE12 cells after Dynasore treatment (Figure 6C-D). Dynasore significantly improved the morphological changes (cell swelling, increase in intracellular contents) induced by NETs (Figure 6E). In addition, Dynasore reduced NETs-induced trimerization of MLKL and inhibited MLKL translocation to plasma membrane-forming pores in MLE12 cells (Figure 6F). We also observed that Dynasore reduced the expression of RIPK3 by Immunofluorescence (Figure 6G). Finally, Dynasore alleviated the phosphorylation of necroptosis-related proteins, RIPK3 and MLKL, induced by NETs activation (Figure 6H-J). All of these data imply that NETs are taken up by AECs through endocytosis and activate the cGAS-STING pathway to trigger the necroptosis of AECs *in vitro*.

### Inhibition of cGAS-STING pathway suppressed NETs-induced necroptosis of AECs in mice

**Figure 7:**
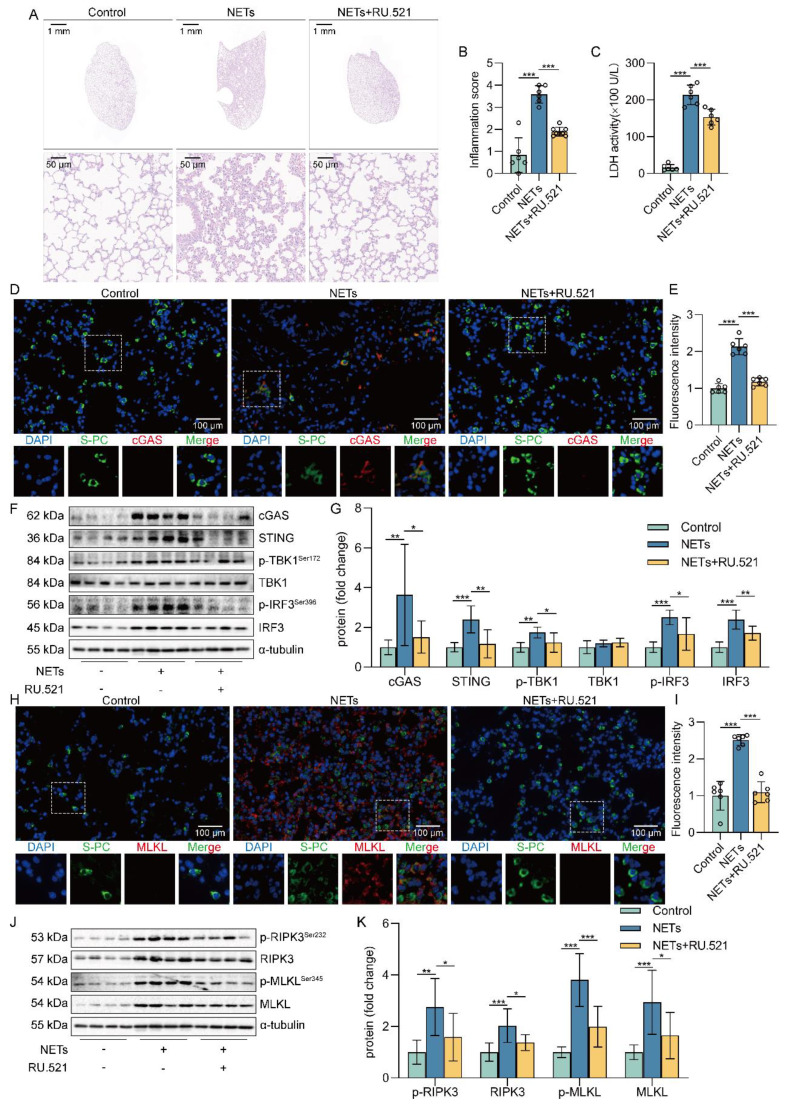
** Inhibition of the cGAS-STING pathway suppresses NETs-induced necroptosis of AECs in mice.** The mice were treated with RU.521 (5 mg/kg) for 2 h before NETs (3 µg/pcs) administration.** A** H&E staining of lung tissue (bar=50 µm). **B** The inflammatory injury score was performed in a double-blind fashion (*n*=6-7). **C** LDH levels in serum (*n*=6). **D-E** Immunofluorescence staining and confocal microscopy were used to determine the localization of cGAS and SP-C (bar=100 µm). Green fluorescence represents alveolar epithelial cell marker protein SP-C, red fluorescence represents necroptosis key protein cGAS, and DAPI blue fluorescence represents the nucleus. **F-G** The expression and phosphorylation levels of cGAS-STING pathway-related proteins were detected in mouse lung tissue by western blot (*n*=8). **H-I** Immunofluorescence staining and confocal microscopy were used to determine the localization of MLKL and SP-C (bar=100 µm). Green fluorescence represents alveolar epithelial cell marker protein SP-C, red fluorescence represents necroptosis key protein MLKL, and DAPI blue fluorescence represents the nucleus. **J-K** The expression and phosphorylation levels of the necroptosis-related proteins RIPK3 and MLKL were detected in mouse lung tissue by western blot (*n*=6). Data are expressed as the mean ± SD. Differences among multiple groups were performed using ANOVA. *P < 0.05, **P < 0.01, and ***P < 0.001.

Based on the above studies, we believe that NETs are taken up by AECs through endocytosis and activate the cGAS-STING pathway to trigger the necroptosis of AECs. To further verify whether NETs trigger necroptosis of AECs in mice by activating the cGAS-STING pathway at the animal level, we used NETs to induce a mouse model of ALI and treated it with RU.521. We found that RU.521 attenuated lung histopathological changes in ALI mice, with thinning of alveolar walls, significant reduction of inflammatory cell infiltration, and less structural destruction of lung tissue (Figure 7A). Then, RU.521 significantly reduced the inflammation score of lung tissue in mice (Figure 7B), suggesting that inhibition of the cGAS-STING pathway could significantly reduce the NETs-induced pathological injury of ALI mice lung tissue. We also observed that RU.521 significantly attenuated LDH levels in the serum of ALI mice (Figure 7C).

In addition, we observed the colocalization of SP-C and cGAS in the lung tissue of NETs-treated mice (Figure 7D-E). The expression of cGAS-STING pathway-related proteins, including cGAS, STING, TBK1, IRF3, and the phosphorylation levels of TBK1 and IRF3, were increased in mice lung tissue treated with NETs (Figure 7F-G). These data suggest that NETs can activate the cGAS-STING pathway of AECs in mice. Moreover, RU.521 significantly reduced the number of necroptosis AECs (SP-C^+^MLKL^+^) in the lung tissue of ALI mice compared with the NETs-treated group (Figure 7H-I). Besides, RU.521 treatment significantly decreased the expression of RIPK3 and MLKL proteins as well as their phosphorylation levels in the lung tissue of mice (Figure 7J-K). These data demonstrate that NETs activate the cGAS-STING pathway to trigger the necroptosis of AECs and promote ALI in mice.

## Discussion

ALI/ARDS is a clinical respiratory disease with high morbidity and mortality 40. Although some progress has been made in the treatment of ALI/ARDS, the efficacy is limited 41. Therefore, it is of great theoretical and clinical significance to investigate the pathogenesis of ALI/ARDS. AECs are the main structural cells of lung tissue, and the occurrence of necroptosis of AECs is a crucial mechanism of diffuse alveolar injury in ALI/ARDS 17, 42, 43. However, the molecular mechanisms that induce necroptosis of AECs during the development of ALI/ARDS remain unclear. In this study, we demonstrated that NETs induced necroptosis of AECs, triggered an inflammatory cascade response, and promoted the occurrence and development of ALI. Mechanistically, we found that NETs taken up by AECs *via* endocytosis can activate the cGAS-STING pathway and then trigger the necroptosis of AECs to promote ALI in mice. In conclusion, our study provides new insights into the molecular mechanisms associated with necroptosis of AECs and their role in the pathogenesis of ALI.

Neutrophil infiltration is one of the most prominent pathological features of ALI/ARDS 22, 44. During the exudative phase of ALI/ARDS, neutrophils, as the earliest immune cells recruited to the site of inflammation, can trap and kill pathogens by releasing NETs 22, 45. NETs are a double-edged sword, which can promote the clearance of pathogens by neutrophils and strengthen the intrinsic immune defense. However, excessive formation or incomplete clearance of NETs may lead to tissue damage and induce uncontrolled inflammation 19, 46. The study found that in sepsis-associated ALI patients and ALI mouse models, serum levels of NETs were found to be positively correlated with disease severity 44. NETs contribute to the development of ALI by inducing lung injury (including alveolar dysfunction, epithelial and endothelial cell damage, and macrophage polarization) directly or indirectly 47-49. Our study found that direct tracheal injection of NETs induced necroptosis of AECs in mice and promoted the occurrence and development of ALI. Our experimental results provide more substantial evidence that excessive release of NETs is one of the key pathogenic mechanisms of ALI. Necroptosis is a regulated form of inflammatory cell death dependent on the activation of RIPK3 and MLKL 50. Necroptosis causes cell swelling, membrane rupture, and release of cytoplasmic contents 51. Our previous study demonstrated that low expression of long optic atrophy protein 1 (L-OPA1) aggravated LPS-induced mitochondrial fragmentation in AECs, impaired mitochondrial function, triggered necroptosis of AECs, and exacerbated ALI 16. Mitochondrial citrate accumulation can recruit cytoplasmic dynamin-related protein 1 (DRP1) through FUN14 domain containing 1 (FUNDC1), exacerbate mitochondrial fragmentation, induce excessive mitochondrial autophagy, and ultimately drive AECs necroptosis, promoting the development of ALI 17. Our study at the cellular level showed that NETs could also induce necroptosis in MLE12 cells, and the results were consistent with the animal levels.

Notably, we observed that NETs-induced necroptosis in MLE12 cells was significantly inhibited by DNase I. DNase I is a DNA degrader that can degrade the dsDNA of NETs 52. RNA-seq analysis also showed that the cytosolic DNA-sensing pathway was activated. The above results suggest that the dsDNA skeleton of NETs may be an essential mediator for NETs to induce necroptosis of AECs. DNA released into the extracellular space often acts as DAMPs binding to the corresponding DNA recognition receptors to promote non-infectious inflammatory responses 53, 54. Studies reported that NETs can activate AIM2 inflammasome in macrophages, promote LPS-induced pyroptosis of alveolar macrophages, and aggravate ALI/ARDS 24, 55. NETs also activate the cGAS-STING pathway in microglia, increasing the release of inflammatory factors and vascular permeability, disrupting the blood-brain barrier, and inducing cerebral hemorrhage 56. In our study, we observed a significant increase in *cgas* gene expression in NETs-treated MLE12 cells, suggesting that NETs released by neutrophils may play a role by binding to intracellular cGAS. The cGAS-STING pathway can recognize intracellular aberrant DNA as well as DNA of invasive pathogens and produce type I interferon to play an immune role 29, 57. Our results showed that NETs could activate the cGAS-STING pathway in MLE12 cells, and DNase I significantly inhibited NETs-induced activation of the cGAS-STING pathway in MLE12 cells. Previous studies reported an unexplored relationship between the key necroptosis protein MLKL and the cGAS-STING pathway 58. Our study observed that inhibition of cGAS-STING pathway activation could inhibit NETs-induced necroptosis in MLE12 cells. We obtained the same results at the animal level. Our study confirms that there is a relationship between the cGAS-STING pathway and necroptosis. NETs can activate the cGAS-STING pathway and then induce necroptosis of AECs. However, it is still unclear whether there is some molecule linking the cGAS-STING pathway and necroptosis, and we will continue to investigate it.

NETs are reticular structures released extracellularly by neutrophils 59, while cGAS are intracellular DNA receptors 36. How do NETs activate intracellular cGAS? Studies reported that AECs have a phagocytic function 60, 61. Yes-associated protein upregulates filopodia formation to promote AEC phagocytosis 61. And IGF-1 promoted the phagocytosis of apoptotic cells by AECs by inducing the expression of PPARγ 62. We hypothesized that NETs could be taken up by AECs into the intracellular compartment and thus bind to cGAS. There are six types of endocytosis, including clathrin-mediated endocytosis (CME), clathrin-independent/dynamin-dependent endocytosis (FEME), clathrin-independent/dynamin-independent endocytosis (CLIC/GEEC), caveolin, macropinocytosis, and phagocytosis 63. High-resolution scanning electron microscopy (SEM) demonstrated that NETs are skeletal networks formed by DNA with a diameter of 15-17 nm and granulin with a diameter of 25 nm, and these fibrous structures can be further aggregated to form 50 nm thread-like structures 64, 65. Based on the size of the diameter of the vesicles formed by the different endocytosis pathways and the properties of the material phagocytosed, as well as the RNA-seq results showing that the expression of clathrin and dynamin protein-related genes were upregulated in NETs-treated MLE12 cells, we speculated that the endocytosis pathway of NETs uptake by AECs might be CME or FEME. We treated MLE12 cells with Dynasore, an inhibitor of CME and FEME, and found that NETs were unable to enter MLE12 cells. Moreover, Dynasore inhibited the activation of the cGAS-STING pathway induced by NETs and attenuated necroptosis in MLE12 cells. Our experimental results verified our conjecture that NETs taken up by AECs through CME or FEME can activate the cGAS-STING pathway and then trigger the necroptosis of AECs. However, it remains unclear whether NETs are taken up by AECs *via* CME or FEME. Furthermore, the activation of specific molecules on the cell membrane of AECs by NETs to trigger endocytosis still requires further investigation. We are committed to continuing our exploration in this area.

Our study has significant advantages. First, we directly treated mice with NETs extracted from mouse bone marrow neutrophils, which more strongly demonstrated that NETs are a vital factor causing ALI. In addition, we used RNA-seq to demonstrate the role of DNA-mediated activation of the cGAS-STING pathway in NETs-induced necroptosis of AECs. However, our study still has some limitations. Firstly, we used tissue immunofluorescence to prove that NETs can cause increased expression of cGAS and MLKL in AECs at the animal level to show that NETs can activate the cGAS-STING pathway and induce necroptosis of AECs. This experiment still cannot fully prove the problem. Secondly, the experimental results showed that DNase I could inhibit the activation of the cGAS-STING pathway induced by NETs and the production of downstream pro-inflammatory factors to a greater extent. Exogenous DNase I could not enter cells, which, to a certain extent, confirmed that dsDNA of NETs was the main cause of activation of the cGAS-STING pathway. However, the influence of mitochondrial DNA still cannot be completely ruled out. Mitochondrial DNA can be released from mitochondria by mitochondrial permeability transition pore (mPTP) and voltage-dependent anion channel (VDAC) 66. Next, we plan to rule out the influence of mitochondrial DNA by using inhibitors of mPTP and VDAC 67. Thirdly, we will use immunomagnetic beads to sort primary mouse AECs to validate the conclusion further. In addition, we only demonstrated that NETs could be taken up by AECs at the cellular level and did not further validate it at the animal level. Next, to further investigate the role of the cGAS-STING pathway in the necroptosis of AECs caused by NETs, cGAS or STING knockout mice may be used in our future study. Finally, we did not study NETs-induced necroptosis of AECs at the clinical level.

**Figure 8:**
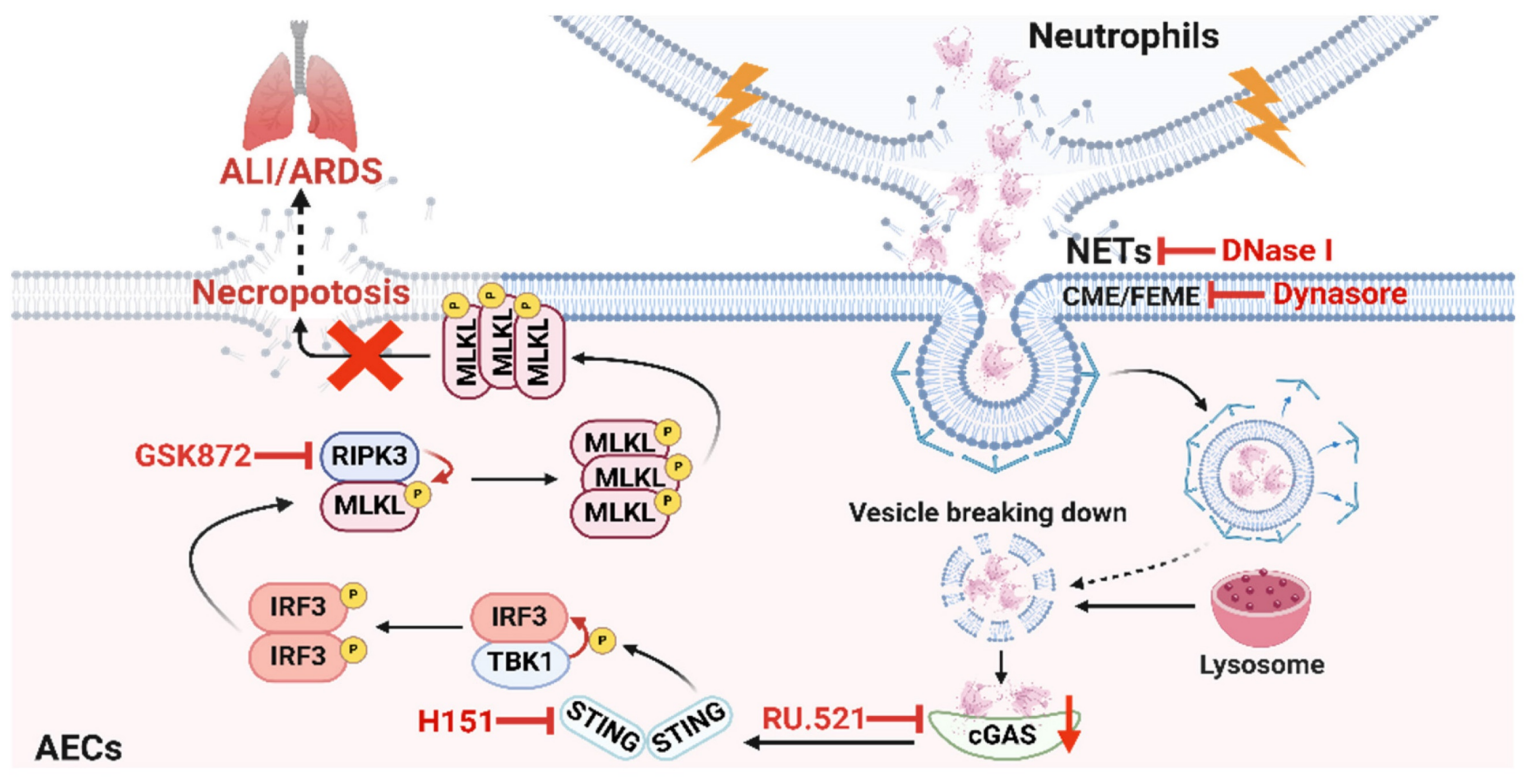
** Schematic illustration.** NETs taken up by AECs *via* endocytosis can activate the cGAS-STING pathway and then trigger the necroptosis of AECs to promote ALI in mice.

In conclusion, our study reveals the critical role of NETs taken up by AECs in activating the cGAS-STING pathway to induce necroptosis of AECs to promote ALI (Figure 8). We conclude that targeting the NETs/cGAS-STING/necroptosis pathway in AECs is an effective strategy for treating ALI.

## Supplementary Material

Supplementary figures.

## Figures and Tables

**Table 1:** Antibody sources and dilutions.

Antibody	Source	Catalog	Dilution ratio
**Primary antibodies for western blot**			
Rabbit anti-MLKL polyclonal antibody	Abcam	Ab172868	1:2000
Rabbit anti-p-MLKL^S345^ monoclonal antibody	Abcam	Ab196436	1:2000
Rabbit anti-RIPK3 polyclonal antibody	Abcam	Ab62344	1:2000
Rabbit anti-p-RIPK3^S232^ monoclonal antibody	Abcam	Ab195117	1:2000
Rabbit anti-p-IRF3^S396^ antibody	ABclonal	AP0623	1:1000
Rabbit-anti-IRF3 antibody	Proteintech	11312-1-AP	1:10000
Rabbit anti-p-TBK1^S172^ antibody	ABclonal	AP1026	1:2000
Rabbit-anti-TBK1 antibody	Proteintech	28397-1-AP	1:2000
Rabbit-anti-STING antibody	Proteintech	19851-1-AP	1:2000
Rabbit-anti-cGAS antibody	CST	31659	1:1000
Anti-α-tubulin monoclonal antibody	Servicebio	GB11200	1:5000
**Secondary antibodies for western blot**			
HRP-conjugated goat anti-rabbit IgG	SAB	L3012-2	1: 5000
Rabbit anti-Goat IgG	SAB	L3042-2	1: 5000
**Primary antibodies for immunofluorescence**			
CoraLite®488-conjugated MLKL antibody	Proteintech	CL488-66675	1: 500
Rabbit anti-MLKL polyclonal antibody	Abcam	Ab172868	1: 200
Rabbit anti-SP-C polyclonal antibody	Boster	A02001	1: 2000
Rabbit anti-RIPK3 polyclonal antibody	Abcam	Ab62344	1: 200
Rabbit-anti-STING antibody	Proteintech	19851-1-AP	1: 200
Rabbit-anti-cGAS antibody	CST	31659	1: 250
Rabbit-anti-IRF3 antibody	Proteintech	11312-1-AP	1: 50
Mouse-anti-NF-κB antibody	Immunoway	YM3111	1: 200
Mouse-anti-MPO antibody	Proteintech	66177-1-Ig	1: 200
Phalloidin-TRITC	Sigma	P1951	1:250
**Secondary antibodies for immunofluorescence**			
FITC goat anti-mouse IgG (H+L)	Abclonal	AS001	1: 400
Rhodamine (TRITC) goat anti-rabbit IgG (H+L)	Abclonal	AS040	1: 400
FITC goat anti-rabbit IgG (H+L)	Abclonal	AS001	1: 400
Polymer-HRP anti-mouse/rabbit universal secondary antibody	AiFang biological	AFIHC001	/

**Table 2:** Sequences of the primers used in this study.

Gene	Forward primer (5'-3')	Reverse primer (5'-3')
*Cgas*	GTTCGGAGATTTAGTCTGTTG	GTGGCTTTATCGGAGTAGGT
*Sting*	CTACATTGGGTACTTGCGGTT	GCACCACTGAGCATGTTGTTATG
*Tbk1*	GATGTGCTTCACCGAATGGT	CGGCTCGTGACAAAGATAGG
*Irf3*	CTACGGCAGGACGCACAGAT	TCAGCAGCTAACCGCAACAC
*Zbp1*	CGTCAGGAAGGCCAAGACAT	TTGGCAATGGAGATGTGGCT
*Aim2*	GTCACCAGTTCCTCAGTTGTG	CACCTCCATTGTCCCTGTTTTAT
*Tlr9*	CAGTTCCTGCCGCTGACTAA	AGGTAGTTGTCTCGGAGGCT
*Ddx41*	AGGTATGCCTTTCCATTGGACACC	ACACCCTTGAGCAGGAGGTA
*Tnfα*	AGCCCCCAGTCTGTATCCTT	CTCCCTTTGCAGAACTCAGG
*Ifnβ*	CGTGGGAGATGTCCTCAACT	CCTGAAGATCTCTGCTCGGAC
*Cxcl10*	CGATGACGGGCCAGTGAGAATG	TCAACACGTGGGCAGGATAGGCT
*Isg56*	TTCCGTAGGAAACATCGCGT	ACATTGTCCTGCCTTCTGGG
*Ccl5*	CACCATATGGCTCGGACACC	TCTGGGTTGGCACACACTTG
*Il-6*	TAGTCCTTCCTACCCCAATTTCC	TTGGTCCTTAGCCACTCCTTC
*β-actin*	TTCCAGCCTTCCTTCTTG	GGAGCCAGAGCAGTAATC

## Abbreviations

AECs: Alveolar epithelial cells
ALI: Acute lung injury
ARDS: Acute respiratory distress syndrome
AIM2: Melanoma 2
BALF: Bronchoalveolar lavage
CitH3: Citrullinated histone 3
cGAS: Cyclic GMP-AMP synthase
cGAMP: Cyclic GMP-AMP
CME: Clathrin-mediated endocytosis
CLIC/GEEC: Clathrin-independent/dynamin-independent endocytosis
DAMPs: Damage-associated molecular molecules
dsDNA: Double-stranded DNA
FEME: Clathrin-independent/dynamin-dependent endocytosis
IRF3: Interferon regulatory factor 3
IFN: Interferon
MLKL: Mixed lineage kinase domain-like protein
mPTP: Mitochondrial permeability transition pore
MPO: Myeloperoxidase
NETs: Neutrophil extracellular traps
NE: Neutrophil elastase
PRRs: Pattern recognition receptors
RIPK3: Receptor-interacting protein kinase 3
STING: Stimulator of interferon genes
TBK1: Tank-binding kinase 1
TNF: Tumor necrosis factor
TLR9: Toll-like receptor 9
VDAC: Voltage-dependent anion channel
ZBP1: Z-DNA binding protein 1
