# Supplementary material for: Neutrophil extracellular traps trigger alveolar epithelial cell necroptosis through the cGAS-STING pathway during acute lung injury in mice
Source: Int J Biol Sci. 2024 Sep 3;20(12):4713–30. doi: 10.7150/ijbs.99456 (PMC11414388; doi:10.7150/ijbs.99456)
Supplement: Supplementary file 1 — Supplementary figures. [file ijbsv20p4713s1.pdf]

Supplementary Figures and Figure Legends

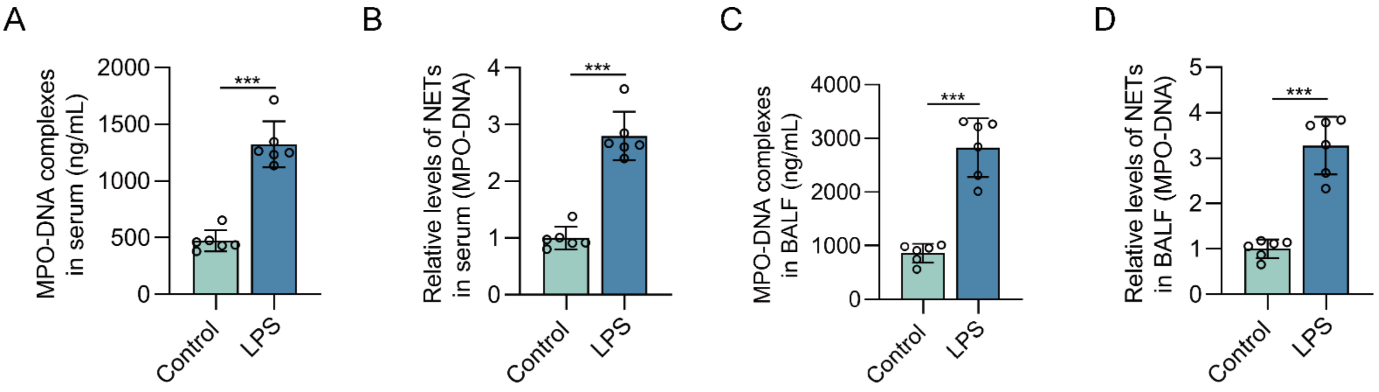

Figure S1. LPS increased the concentration of NETs in serum and BLAF of mice. C57BL/6J mice were intratracheally injected with LPS (5 mg/kg). Mice were killed 12 h after injection of LPS, and serum and bronchoalveolar lavage (BALF) were collected. An ELISA kit was used to measure the amount of MPO-DNA in serum and BALF of mice. **A** MPO-DNA complexes in serum (*n*=6). **B** Relative levels of NETs in serum (MPO-DNA) (*n*=6). **C** MPO-DNA complexes in BLAF (*n*=6). **D** Relative levels of NETs in BLAF (MPO-DNA) (*n*=6). Data are expressed as the mean ± SD. Comparisons between the two groups were made using an unpaired *t*-test. ***$P < 0.001$.

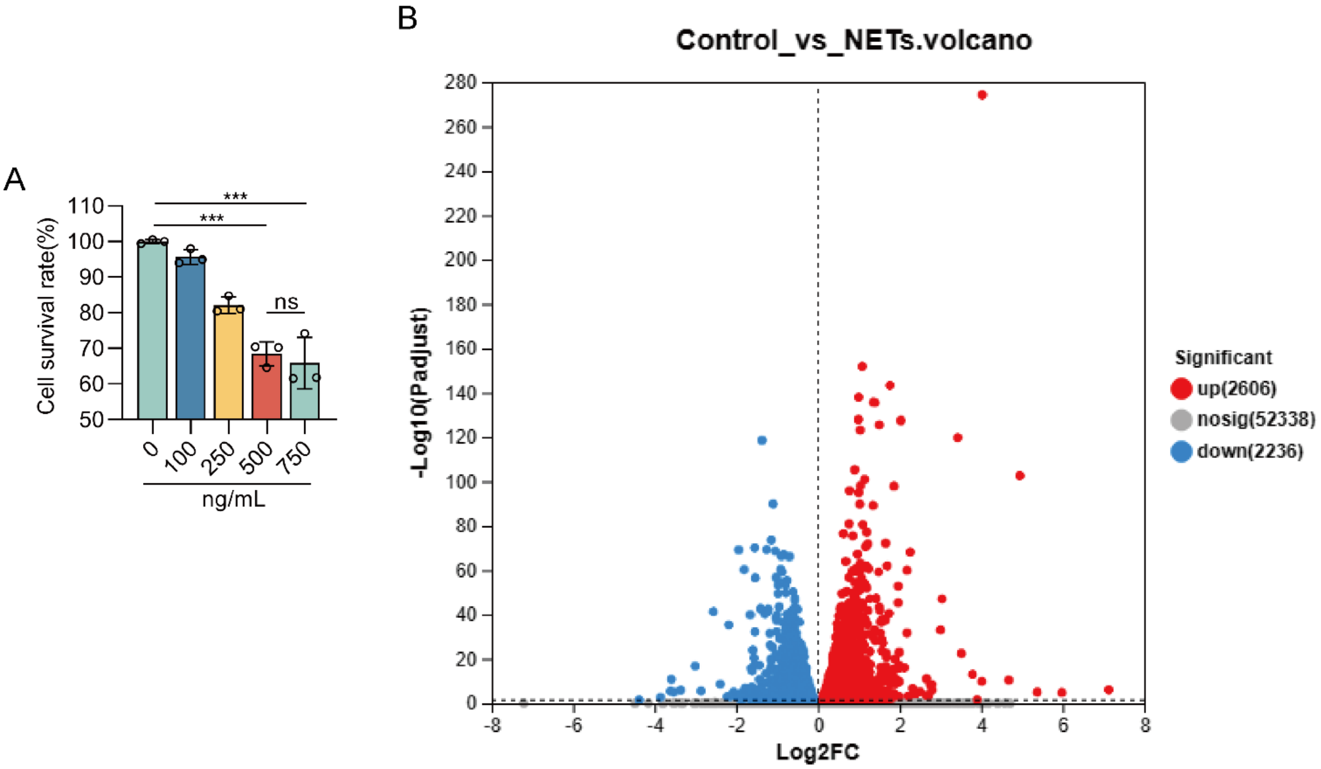

Figure S2. NETs can cause damage to AECs. A MLE12 cells were treated with different concentrations of NETs (100, 250, 500, and 750 ng/mL) for 12 h. MLE12 cell viability was evaluated by CCK-8 assay (n=3). **B** Significantly changed RNAs were visualized in volcano plots. Red and blue dots indicate up-regulated and downregulated genes, respectively. Differentially expressed genes in NETs-treated MLE12 cells compared with normal cells. Data are expressed as the mean ± SD. Differences among multiple groups were performed using ANOVA. ***$P < 0.001$.

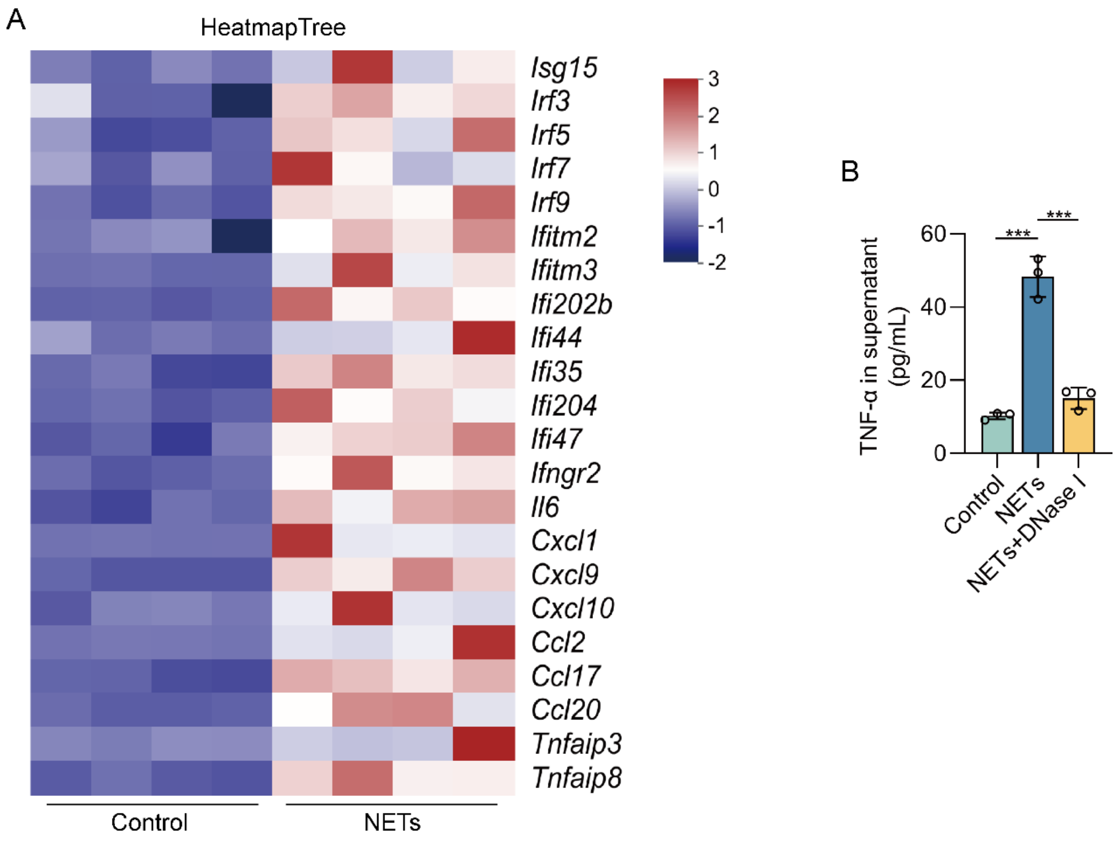

Figure S3. NETs can cause upregulation of pro-inflammatory factor gene expression in AECs. MLE12 cells were treated with NETs (500 ng/mL) for 12 h after DNase I (10 μg/mL) intervention 30 min earlier. **A** Heatmap analysis of RNA sequencing for pro-inflammatory factor gene expression in MLE12 cells as indicated. **B** TNF-α production in the supernatant (n=3). Data are expressed as the mean ± SD. Differences among multiple groups were performed using ANOVA. ***$P < 0.001$.

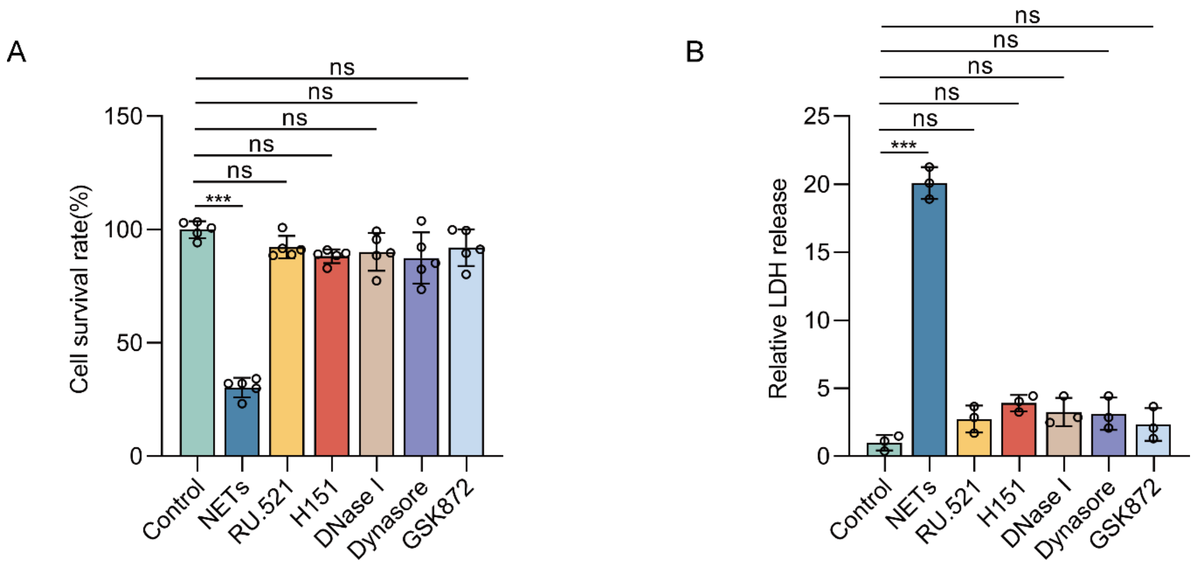

Figure S4. Drug treatment alone had no significant effect on the viability of AECs *in vitro*. MLE12 cells were treated with RU.521 (10 μM), H151 (5 μM), DNase I (10 μg/mL), Dynasore (80 μM), or GSK872 (10 μM) for 12 h. **A** MLE12 cell viability was evaluated by CCK-8 assay (*n*=5). **B** Evaluation of MLE12 cell mortality by LDH release assay (*n*=3). Data are expressed as the mean ± SD. Differences among multiple groups were performed using ANOVA. ***$P < 0.001$.
